# Synergistic Enhancement of Carboplatin Efficacy through pH-Sensitive Nanoparticles Formulated Using Naturally Derived *Boswellia* Extract for Colorectal Cancer Therapy

**DOI:** 10.3390/pharmaceutics16101282

**Published:** 2024-09-30

**Authors:** Sherif Ashraf Fahmy, Nada K. Sedky, Hatem A. F. M. Hassan, Nour M. Abdel-Kader, Noha Khalil Mahdy, Muhammad Umair Amin, Eduard Preis, Udo Bakowsky

**Affiliations:** 1 Department of Chemistry, School of Life and Medical Sciences, University of Hertfordshire Hosted by Global Academic Foundation, R5 New Garden City, New Administrative Capital, Cairo 11835, Egypt; 2 Department of Pharmaceutics and Biopharmaceutics, University of Marburg, Robert-Koch-Str. 4, 35037 Marburg, Germany; muhammad.umairamin@pharmazie.uni-marburg.de (M.U.A.); eduard.preis@pharmazie.uni-marburg.de (E.P.); 3 Department of Biochemistry, School of Life and Medical Sciences, University of Hertfordshire Hosted by Global Academic Foundation, R5 New Garden City, New Administrative Capital, Cairo 11835, Egypt; nadasedky22@gmail.com (N.K.S.); nmohamed@gaf.edu.eg (N.M.A.-K.); 4 Medway School of Pharmacy, Universities of Kent and Greenwich, Chatham Maritime, Kent ME4 4TB, UK; 5 Department of Pharmaceutics and Industrial Pharmacy, Faculty of Pharmacy, Cairo University, Cairo 11562, Egypt; nkmahdy@gmail.com; 6 Department of Biochemistry, Faculty of Science, Ain Shams University, Cairo 11566, Egypt

**Keywords:** colorectal cancer, chemotherapy, nanomedicine, nanoprecipitation, pH-sensitive release

## Abstract

Carboplatin (Cp) is a potent chemotherapeutic agent, but its effectiveness is constrained by its associated side effects. Frankincense, an oleo-gum resin from the *Boswellia sacra* tree, has demonstrated cytotoxic activity against cancer cells. This study explored the synergistic potential of nanoparticles formulated from *Boswellia sacra* methanolic extract (BME), to enhance the therapeutic efficacy of Cp at reduced doses. Nanoparticles were prepared via the nanoprecipitation method, loaded with Cp, and coated with positively charged chitosan (CS) for enhanced cell interaction, yielding Cp@CS/BME NPs with an average size of 160.2 ± 4.6 nm and a zeta potential of 12.7 ± 1.5 mV. In vitro release studies revealed a pH-sensitive release profile, with higher release rates at pH 5.4 than at pH 7.4, highlighting the potential for targeted drug delivery in acidic tumor environments. In vitro studies on HT-29 and Caco-2 colorectal cancer cell lines demonstrated the nanoformulation’s ability to significantly increase Cp uptake and cytotoxic activity. Apoptosis assays further confirmed increased induction of cell death with Cp@CS/BME NPs. Cell-cycle analysis revealed that treatment with Cp@CS/BME NPs led to a significant increase in the sub-G1 phase, indicative of enhanced apoptosis, and a marked decrease in the G1-phase population coupled with an increased G2/M-phase arrest in both cell lines. Further gene expression analysis demonstrated a substantial downregulation of the anti-apoptotic gene Bcl-2 and an upregulation of the pro-apoptotic genes Bax, PUMA, and BID following treatment with Cp@CS/BME NPs. Thus, this study presents a promising and innovative strategy for enhancing the therapeutic efficacy of chemotherapeutic agents using naturally derived ingredients while limiting the side effects.

## 1. Introduction

With over 1.85 million new diagnoses of colorectal cancer (CRC) each year and a mortality rate of 46%, CRC ranks as the third most common cause of cancer-related deaths among both genders [1]. Men and women of all racial and ethnic backgrounds are susceptible to CRC. In 2020, CRC accounted for 515,637 deaths among men and 419,536 fatalities among women globally [2]. CRC can be classified into five stages based on the depth of tumor invasion, with stage 0 representing the earliest stage and stage IV the most advanced. At stage 0, CRC is often managed through surgical resection of the tumor. A combination of chemotherapy and surgical interventions is typically employed for the more advanced stages. Platinum-based drugs are commonly utilized in CRC treatment due to their significant anticancer efficacy. However, platinum-based drugs are associated with considerable adverse effects and drug resistance, which may be attributed to reduced cellular uptake, increased detoxification, and enhanced DNA repair mechanisms [3,4,5].

Cp, a second-generation platinum-based drug, is a broad-spectrum antitumor agent characterized by a platinum atom complexed with a cyclobutane-dicarboxylate backbone and two ammonia groups. Intracellularly, Cp is activated to form reactive platinum complexes that bind to the nucleophilic groups on DNA. This binding results in both intrastrand cross-linking and DNA–protein cross-linking, ultimately leading to apoptosis and reduced cell proliferation. Various nanoparticles, including supramolecular structures, metal and metal oxide NPs, polymeric NPs, dendrimers, and lipid-based NPs, have been employed to encapsulate platinum drugs, enhancing their anticancer efficacy while mitigating their undesirable side effects [6,7,8].

Frankincense is an oleo-gum resin extracted from the trunks of *Boswellia sacra* trees. It was previously reported that frankincense can selectively target cancerous cells while exerting no cytotoxic effects on healthy cells [9,10]. Boswellic acids are among the principal constituents of frankincense and have been reported to possess significant antitumor properties [11,12]. Using nanoformulations to deliver therapeutic agents could offer significant advantages by enhancing therapeutic activity while reducing adverse effects [13,14,15,16,17]. Nanoprecipitation is a simple and effective method for preparing nanoparticles through solvent/nonsolvent precipitation, where the terms ‘solvent’ and ‘nonsolvent’ refer to the solubility of the components that form the nanoparticles. This technique has been successfully employed to prepare a wide range of nanoparticles from diverse starting materials due to its uncomplicated setup, low cost, and minimal environmental impact [18]. *Boswellia* extract was previously converted into nanoparticles using solvent shifting, specifically nanoprecipitation in an anti-solvent, for application in cancer therapy [19].

Extended drug release from nanocarriers is required to enhance the antitumor efficacy of platinum-based drugs [3]. Chitosan (CS) is a positively charged, natural, biocompatible, and biodegradable polymer that can be used for the surface modification of nanoparticles through coating. Coating nanocarriers with CS has been shown to extend drug release rates, improve stability, enhance cellular drug uptake, and reduce drug leakage from the nanoparticles. Coating of nanoparticles’ surfaces with CS can be achieved via electrostatic interactions between the positively charged chitosan and the negatively charged nanocarriers [3]. In this study, the oleo-gum resin, a major active ingredient from *Boswellia sacra*, was extracted using methanol. The *Boswellia sacra* methanol extract (BME) was then characterized with LC/ESI-MS/MS. Subsequently, the BME was nanosized using the solvent-shift approach, loaded with Cp, and coated with CS to produce Cp@CS/BME NPs. The yielded nanoformulations were characterized to determine their physicochemical properties. Furthermore, the cellular uptake of free Cp and Cp@CS/BME NPs, as well as the cytotoxic and apoptotic effects following incubation with HT-29 and Caco-2 colorectal cancer cell lines, was assessed.

## 2. Materials and Methods

### 2.1. Materials

Oleo-gum resins were collected from *Boswellia sacra* plants cultivated in Oman. Dialysis bags with a molecular weight cutoff of 10–12 kDa were obtained from Solarbio (Beijing, China). RPMI medium, penicillin, streptomycin, fetal bovine serum, Tris buffer, and the sulforhodamine B (SRB) assay kit were acquired from Lonza (Basel, Switzerland). The Annexin V–FITC apoptosis detection kit was obtained from Abcam Inc. (Cambridge, UK). The QIAamp Viral RNA Mini Kit and SYBR Green PCR Kit were obtained from Qiagen (Hilden, Germany). The RevertAid RT Kit was purchased from Thermo Fisher Scientific (Waltham, MA, USA). RT-qPCR primers were obtained from Metabion International AG (Bavaria, Germany). All other reagents were procured from Sigma-Aldrich (Burlington, VT, USA).

### 2.2. Extraction and Chemical Characterization of the Major Chemical Components of Boswellia Sacra Resin

The major chemical components of *Boswellia sacra* oleo-gum resin (BSR) were extracted using methanol to obtain the BME, following a previously reported method, with some modifications [3]. The BSR pieces were ground, and 100 mg of the ground material was added to 300 mL of methanol. Then, the mixture was stirred at room temperature for 24 h in a Schott bottle. The mixture was filtered twice, and the methanol was evaporated under vacuum using a rotary evaporator. The resulting BME powder was purified by redissolving it in methanol, and the solution was filtered through a syringe filter (pore size: 0.22 µm). The clear solution was then placed in an oven at 60 °C until the BME powder was completely dry and the solvent was evaporated. The primary chemical constituents of the BME were identified using liquid chromatography–electrospray ionization–tandem mass spectrometry (LC/ESI-MS/MS) with an X500R LC-QTOF mass spectrometer (SCIEX, Framingham, MA, USA). Separation was performed on an Inertsil C18 column (25 cm × 4.6 mm × 5 µm). The mobile phases comprised A: (80:20) methanol and B: 0.1% formic acid. The gradient elution was programmed as follows: 3% B from 0 to 5 min, 3–90% B from 5 to 18 min, 90% B from 18 to 23 min, 90–3% B from 23 to 27 min, and 3–1% B from 27 to 30 min. The sample volume was 6 µL, and the flow rate was set at 1.0 mL/min. Negative ionization mode was used for the MS/MS analysis, with SWATH scanning from 50 to 1000 Da, and the following parameters: curtain gas at 30 psi, ion spray voltage at 5000 V, source temperature at 500 °C, ion source gases 1 and 2 at 50 psi each, declustering potential at 80 V, and collision energy at 10 V. Compound identification was performed by comparing the results to the NIST Library, a built-in reference library.

### 2.3. Preparation of Cp@CS/BME NPs

BME nanoparticles (BME NPs) were prepared using the solvent-shift method, as described previously, with few modifications [19]. Briefly, BME and Cp (0.2%) were dissolved in ethanol, forming the organic phase. The organic phase was then added dropwise to the aqueous phase, consisting of deionized water or polyvinyl alcohol at various concentrations, while continuously stirring. The obtained dispersion was then subjected to ultrasonication for 10 min, followed by overnight stirring on a magnetic stirrer to evaporate the ethanol. Subsequently, the obtained Cp-loaded BME nanoparticles (Cp@BME NPs) were coated with CS by adding a CS solution in 1% (*v*/*v*) glacial acetic acid (1:10) dropwise to the nanoparticle dispersion while stirring continuously, yielding Cp@CS/BME NPs. This was followed by 10 min of sonication in a bath sonicator and an additional 2 h of stirring using a magnetic stirrer [20]. The prepared nanoparticles were stored at 4 °C until further tests.

### 2.4. Size, Polydispersity Index (PDI), and Zeta Potential Analysis

The engineered nanoparticles were characterized in terms of average particle size, PDI, and zeta potential using a ZS Malvern Zetasizer equipped with a 10 mW HeNe laser (Malvern Instruments, Worcestershire, UK) at 25°C [21,22,23]. All measurements were performed in triplicate, and the results are presented as the mean ± standard deviation (SD).

### 2.5. Transmission Electron Microscopy

The morphological topography of the NPs was examined using high-resolution transmission electron microscopy (JEOL JEM-2100, Musashino, Akishima, Tokyo, Japan) operating at 140 kV. The nanoparticles were diluted 1:2 with deionized water, stained with 2% aqueous phosphotungstic acid, and finally dried over a carbon-coated copper 200-mesh grid for imaging.

### 2.6. Entrapment Efficiency (EE)

The EE% of Cp incorporated within the nanoparticles was carried out following a previously reported procedure [20]. Free Cp was isolated via centrifugation of the dispersion at 12,000× *g* rpm and 4 °C for 3 h (Hermle Z 326 K, Labortechnik GmbH, Wehingen, Germany). The unencapsulated Cp was then quantified using HPLC. The EE% was calculated using Equation (1):

(1) $$EE\%=\frac{Initial\;amount\;of\;Cp-the\;amount\;of\;free\;Cp}{Initial\;amount\;of\;Cp}\times 100\%$$

### 2.7. In Vitro Drug Release Studies

The membrane dialysis method was utilized to evaluate the release of Cp from the BME nanoparticles, simulating the nanoformulation’s release behavior in bodily fluids [23]. A specific quantity of nanoparticles encapsulating a fixed dose of Cp was mixed with 1 mL of PBS at pH 5.4 and pH 7.4, representing tumor pH and physiological pH, respectively. The dispersion was then loaded into dialysis bags with an average molecular weight cutoff of 10–12 kDa (Solarbio, Beijing, China). To monitor Cp release during a 48 h period, the dialysis bags were sealed, submerged in 40 mL of dissolution medium, and placed on a magnetic stirrer set to gently agitate at 150 rpm at a controlled temperature of 37 ± 1 °C. Samples of 200 μL were collected in triplicate at specific timepoints (0, 0.5, 1, 2, 4, 6, 8, 12, 18, 24, and 48 h). Fresh medium at the same temperature was added after each sampling to maintain a consistent dissolution medium volume. The drug concentration was then quantified using HPLC, as outlined in our previously published method [24]. The release % was calculated using Equation (2):

(2) $$Release\;Efficiency\;\%=\frac{Amount\;of\;released\;Cp}{Initial\;amount\;of\;loaded\;Cp}\times 100\%$$

### 2.8. Release Kinetics

The release kinetics of Cp from the nanoformulation was determined using the release percentages obtained from in vitro release studies and mathematical kinetics models, as previously described in [25,26]. The kinetics models, including zero-order, first-order, Korsmeyer–Peppas, Higuchi, and Hixson–Crowell models, were applied utilizing Equations (3)–(7):

(3) $$C=k_{0}\;t$$

(4) $$\ln C=\ln C_{0}-k_{1\;}t$$

(5) $$C=k_{k}\;t^{n}$$

(6) $$C=k_{H}\;\sqrt{t}$$

(7) $$\sqrt[{3}]{W_{0}}-\sqrt[{3}]{W_{t}\;}=K_{ß}\;t$$

where *C* represents the cumulative percentage of drug released at time *t*, *K*_0_ denotes the rate constant for the zero-order model, *K*_1_ refers to the rate constant for the first-order model, *K_H_* represents the Higuchi constant, *K_K_* is the Korsmeyer–Peppas constant, *n* is the exponent characterizing the specific diffusion mechanism, *W*_0_ indicates the initial drug amount in the system, *W_t_* is the remaining drug amount at time *t*, and *K_ß_* represents the Hixson–Crowell release constant.

### 2.9. Cell Culture

The colorectal cancer cell lines HT-29 and Caco-2 and the normal human colon cell line CCD 841 CoN were obtained from the American Type Culture Collection (ATCC). The cells were cultured in RPMI medium supplemented with 100 µg/mL streptomycin, 100 units/mL penicillin, and 10% heat-inactivated fetal bovine serum. The culture flasks were incubated in a humidified atmosphere with 5% (*v*/*v*) CO_2_ at 37 °C.

### 2.10. Cell Viability

The SRB viability assay was employed to evaluate the cytotoxicity of BME, BME NPs, Cp, and Cp@CS/BME NPs against both colorectal carcinoma and normal colon cell lines. For each treatment, six different logarithmic concentrations ranging from 0.01 to 1000 µg/mL were prepared. The assay was conducted in 96-well cell culture plates, where 100 µL of cell suspension (containing 5 × 10^3^ cells) was cultured in a complete medium in each well for 24 h. The cells were then exposed to 100 µL of medium containing varying concentrations of the different treatments for an additional 48 h. The culture medium was removed, and the cells were fixed with 150 µL of 10% trichloroacetic acid for 1 h at 4 °C. Following fixation, the cells were washed five times with distilled water to remove excess trichloroacetic acid. Cellular proteins were stained for 10 min in the dark with 70 µL of 0.4% (*w*/*v*) SRB solution, followed by three washes with 1% acetic acid to remove any excess stain. The plates were then air-dried overnight. The stained proteins were dissolved in 150 µL of 10 mM Tris, and the concentration was measured spectrophotometrically at 540 nm using a BMGLABTECH^®^ FLUOstar Omega microplate reader (Ortenberg, Germany).

### 2.11. Apoptosis Assay

The Annexin V–FITC apoptosis detection kit (Abcam Inc., Cambridge Science Park, Cambridge, UK) was utilized to quantify the percentages of apoptotic and necrotic cells within the cellular population following exposure to Cp@CS/BME NPs. Both HT-29 and Caco-2 cells were cultured for 48 h in a complete medium containing the test compounds. Trypsinization was performed to collect a total of 10^5^ cells for each treatment. The collected cells were pre-washed twice with ice-cold PBS (pH 7.4) before staining. Cell labeling was conducted by incubating the cells with 0.5 mL of Annexin V–FITC/PI solution in the dark for 30 min, followed by data acquisition using an ACEA Novocyte™ flow cytometer. FITC and PI fluorochromes were detected using FL1 (λ_ex/em_ 488/530 nm) and FL2 (λ_ex/em_ 535/617 nm), respectively. A total of 12,000 events were collected for each sample, and the data were analyzed using ACEA Novo Express™ software 1.6.2 (ACEA Biosciences Inc., San Diego, CA, USA), with the results sorted into quadrants representing FITC and PI interactions with the cells.

### 2.12. Cellular Uptake of Cp

HT-29 and Caco-2 colorectal cancer cells were seeded at a density of 4 × 10^5^ cells per T25 flask and incubated overnight. The medium was then carefully aspirated without disturbing the cell monolayer, and fresh media containing 30 µg/mL of either Cp or Cp@CS/BME NPs were added. The treated flasks were incubated at 37 °C with 5% CO_2_ for 6 h. Following incubation, the media and cell pellets were collected and analyzed using HPLC. For the HPLC analysis, 0.5 mL of acetonitrile was added to each pellet, which was then sonicated for 15 min and centrifuged at 5000 rpm for 5 min. The supernatant was transferred into an autosampler vial without further dilution, filtered through a PTFE syringe filter (0.45 µm), and injected into the HPLC system. The HPLC conditions are detailed in our previous work [24]. The % uptake was calculated using Equation (8):

(8) $$Uptake\%=\frac{Amount\;of\;Cp\;determined\;inside\;the\;cells}{Initial\;amount\;of\;Cp\;added}\times 100\%$$

### 2.13. Cell-Cycle Analysis

The effects of the newly synthesized preparation on the cell cycle of both HT-29 and Caco-2 cells were assessed using flow cytometry. Cells were treated with the Cp or Cp@CS/BME NPs for 48 h. Trypsinization was performed to collect a total of 10^5^ cells for the assay. The cells were washed twice with ice-cold PBS (pH 7.4) before fixation with 2 mL of ice-cold 60% ethanol for 1 h at 4 °C. The cells were rewashed twice with PBS and then suspended and stained in the dark for 20 min with 1 mL of PBS containing RNase A (50 µg/mL) and propidium iodide (PI) (10 µg/mL). The DNA content was analyzed using FL2 (λ_ex/em_ 535/617 nm) on an ACEA Novocyte™ flow cytometer, and the ACEA NovoExpress™ software (ACEA Biosciences Inc., San Diego, CA, USA) was used to estimate the percentage of cells in each phase of the cell cycle.

### 2.14. Gene Expression Analysis

Gene expression analysis for the target genes was conducted following 48 h of cellular exposure to the nanoformulation. The apoptotic genes (BID, BIK, and Noxa) and the anti-apoptotic gene (Bcl-2) were examined using RT-qPCR, as described in our previous work [5]. β-Actin was used as the housekeeping gene for data normalization. Briefly, mRNA extraction was performed according to the manufacturer’s instructions using the QIAamp Viral RNA Mini Kit. The DNA concentration and purity were assessed using a NanoDrop Spectrophotometer. Subsequently, cDNA synthesis was carried out using the RevertAid RT Kit. Gene quantification was performed with the SYBR Green PCR Kit, using the primers listed in Table 1 and the Rotor-Gene Q thermal cycler (Qiagen). Finally, the relative expression of each selected gene was calculated using the 2^−ΔΔCt^ method.

### 2.15. Statistical Analysis

The statistical analysis was performed using GraphPad Prism 9 (USA). The experimental results are presented as the mean ± standard deviation (SD) of three independent experiments. For multiple comparisons, one-way ANOVA followed by Tukey’s post hoc test was employed, while *t*-tests were used to compare the means of two groups. Statistical significance was determined at a *p*-value ≤ 0.05.

## 3. Results and Discussion

### 3.1. LC/ESI-MS-MS Analysis of the Boswellia Methanol Extract

**Figure 1:**
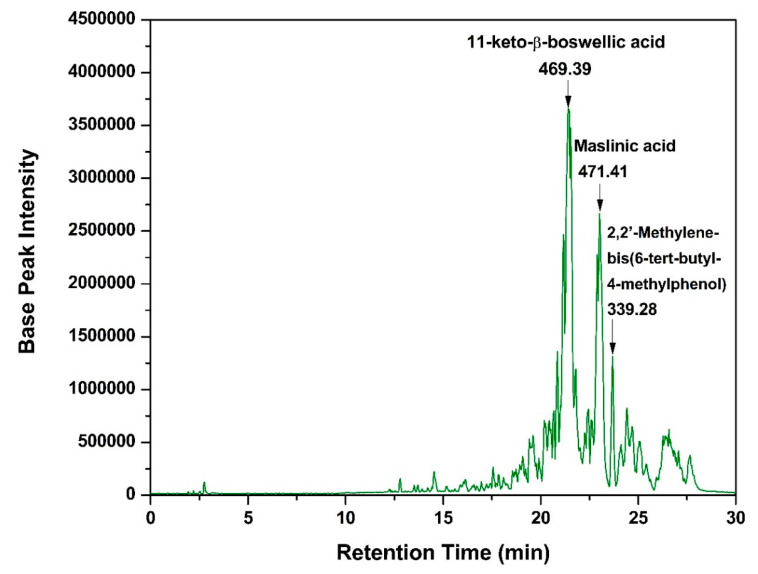
LC-ESI-MS/MS ion chromatogram (in the negative ion mode) of the *Boswellia sacra* oleo-gum resin methanolic extract.

The chemical profile of the BME was analyzed using LC-MS/MS in negative high-resolution ESI mode. The representative base peak chromatograms of the methanolic extract are presented in Figure 1. Data processing for feature detection resulted in 3803 detected peaks, of which 35 were identified and confirmed based on accurate molecular mass and MS/MS fragment ions, compared with data from the NIST Library. The confirmed peaks, along with their assignments, retention times, m/z of detected molecular ions, molecular formulae, product ions (MS/MS), and compound classes, are shown in Table 2. As demonstrated in Figure 1, the BME contained three compounds with the highest intensities: 11-keto-beta-boswellic acid, maslinic acid, and 2,2′-methylene-bis(6-tert-butyl-4-methylphenol). These compounds have previously been demonstrated to exert strong antitumor activity [27,28]. These findings confirm the potential potent antitumor effect of the BME.

The identified compounds were categorized into several classes. Four triterpenes were found: madecassic acid, maslinic acid, 11-keto-beta-boswellic acid, and 3-acetyl-11-keto-beta-boswellic acid. In addition, three phenols were identified, including 2-(2-hydroxyethoxy)phenol, 2′-hydroxy-4′-methoxyacetophenone, and 2,2′-methylene-bis(6-tert-butyl-4-methylphenol). Two phenolic acids were noted, with neochlorogenic acid appearing twice. Two fatty acids were also detected: 7,7-dimethyl-(5Z,8Z)-eicosadienoic acid and cis-4,10,13,16-docosatetraenoic acid. Furthermore, two bile acids were identified: trihydroxycholestanoic acid and 3β,7α-dihydroxy-5-cholestenoic acid. Moreover, one disaccharide, D-(+)-trehalose, was found. One phospholipid, tridecanoyl-sn-glycero-3-phosphate, and one hydroxycoumarin, 6-fluoro-4-hydroxycoumarin, were also identified. The analysis also revealed one steroid, 4-androsten-17β-ol-3-one sulfate, one alkaloid, N-2-hydroxyethylpiperazine, and one eicosanoid, thromboxane B3. Additionally, one monoterpene, (+)-trans-chrysanthemic acid, and one flavonoid, genkwanin, were present. The acids that did not fit into any of the above categories were classified as organic acids. Seven organic acids were identified, including quinic acid, ureidosuccinic acid, methylmalonic acid, oxalacetic acid, a gabapentin-related compound E, 3-phenylbutyric acid, and 4-chloro-alpha-(4-chlorophenyl)-benzeneacetic acid.

### 3.2. Average Diameters, PDI, Zeta Potential, Morphology, and Entrapment Efficiency (EE%)

**Figure 2:**
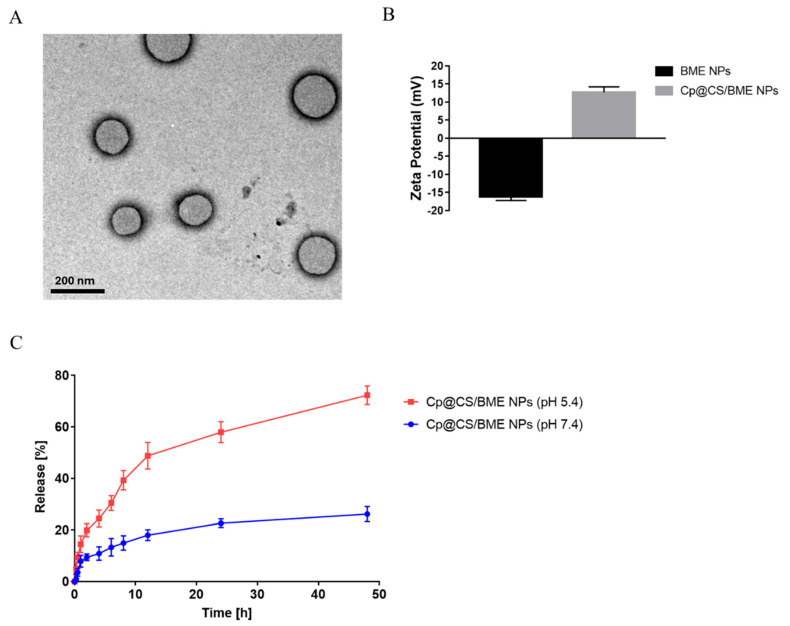
Physicochemical characterization of Cp@CS/BME NPs: (**A**) TEM image illustrating the morphology of Cp@CS/BME NPs; scale bar = 200 nm. (**B**) Zeta potential of BME NPs and Cp@CS/BME NPs. (**C**) In vitro release profile of Cp from Cp@CS/BME NPs into acetate buffer (pH 5.4) and phosphate buffer (pH 7.4). Data are presented as the mean ± SD; n = 3.

The physicochemical characterization of the formulated BME NPs and Cp@CS/BME NPs highlighted the impact of CS coating and drug loading on the nanoparticles’ properties (Table 3 and Figure S1). The BME NPs exhibited an average particle size of 120.1 ± 5.1 nm, whereas the Cp@CS/BME NPs showed a larger size of 160.2 ± 4.6 nm. This increase in particle size might have been due to the additional CS layers and the incorporated Cp [29,30]. In addition, the BME NPs and Cp@CS/BME NPs demonstrated PDI values of 0.11 ± 0.03 and 0.14 ± 0.04, respectively. The low PDI values reflected a relatively narrow size distribution, indicating that the formulation process maintained a good degree of homogeneity even after coating and drug loading. The TEM images further demonstrated the spherical morphology of the Cp@CS/BME NPs (Figure 2). The small particle size of the formulated Cp@CS/BME NPs could allow for efficient biodistribution and cellular uptake [31].

The zeta potential analysis (Figure 2B) revealed distinct differences in surface charge between the uncoated BME NPs and Cp-loaded BME nanoparticles coated with CS (Cp@CS/BME NPs). The BME NPs exhibited a zeta potential of −16.1 ± 1.1 mV that was, conversely, increased to 12.7 ± 1.5 mV upon CS coating and the formation of Cp@CS/BME NPs. This remarkable increase in the surface charge can be attributed to the presence of the positively charged ammonium groups of CS. Our findings indicate the successful coating of the nanoparticles with the cationic polymer CS via electrostatic deposition [29]. The positive zeta potential possessed by Cp@CS/BME NPs could facilitate the interactions with negatively charged cell membranes and improve the nanoparticles’ cellular uptake [32,33,34].

The Cp@CS/BME NPs demonstrated a high EE% of 86.5 ± 2.8%, indicating that the formulation process was effective in incorporating Cp into the nanoparticles. A high EE% is crucial for maximizing the therapeutic payload delivered to the target site, thereby potentially enhancing the anticancer efficacy of the formulation. The successful encapsulation of Cp within the CS-coated BME NPs suggests an efficient formulation strategy and supports the potential of this system to deliver therapeutic doses of Cp effectively. These findings collectively suggest that Cp@CS/BME NPs could offer high drug-loading capacity and improved cellular interaction.

### 3.3. Cp Release from BME NPs In Vitro

The release profile of Cp from BME NPs was evaluated at pH 5.4, simulating the acidic microenvironment of tumor tissues, and pH 7.4, representing the physiological pH of normal bodily fluids [35]. The in vitro release study, conducted using a membrane dialysis technique, demonstrated distinct release behaviors under these conditions, as shown in Figure 2. At pH 5.4, a significantly higher release of Cp from the BME NPs was observed compared to pH 7.4. The release of Cp at pH 5.4 exhibited a rapid initial release phase, reaching approximately 40% within the first 8 h. This was followed by a sustained release, reaching nearly 75% at the end of the 48 h period, owing to the protonated amino groups and the swelling manner of CS. Conversely, at pH 7.4, the release of Cp was considerably slower, with only about 25% released over the same 48 h period, suggesting that most of the drug remained entrapped, surrounded by the BME NPs matrix, as the integrity of the CS coating was preserved at physiological pH [36].

Our findings agreed very well with similar previous studies [37,38,39]. The difference in release profiles can be attributed to the acidic pH environment, which could enhance the degradation of certain types of nanocarrier systems, thereby promoting the release of the encapsulated drug [40,41]. The rapid release observed at pH 5.4 suggests that the BME NPs are particularly responsive to acidic conditions, making them potentially effective in targeting tumor tissues, where the pH is typically lower than in normal tissues. These results indicate that the BME NPs could provide a controlled release of Cp, with the potential for higher drug availability in the tumor microenvironment due to the accelerated release at acidic pH [42]. This pH-sensitive release characteristic could be harnessed in targeted cancer therapy, where minimizing drug release in normal tissues and enhancing release in the tumor microenvironment can reduce side effects and improve therapeutic outcomes [43].

### 3.4. Release Kinetics of Cp from BME NPs

**Figure 3:**
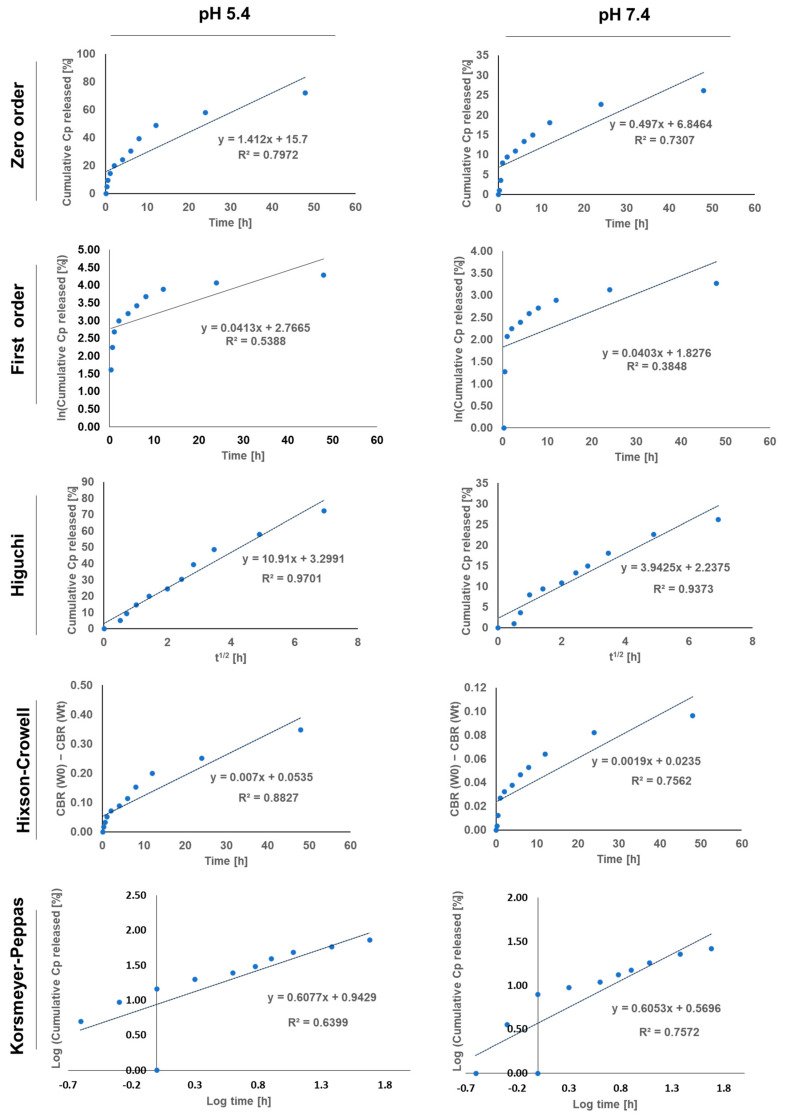
The release kinetics of Cp from BME NPs at pH 5.4 and 7.4, were determined using mathematical kinetics models.

The release kinetics of Cp BME NPs were analyzed using several mathematical models, including zero-order, first-order, and Higuchi models, to understand the mechanism and rate of drug release under different pH conditions (Figure 3) [44]. The Higuchi model exhibited the highest correlation coefficient (R²) for both pH conditions, indicating that diffusion was the primary mechanism governing the release of Cp from the BME NPs (Table 4). This model suggests that the drug release was mainly controlled by the diffusion rate through the nanoparticle matrix, consistent with the observed steady release rates over time [45]. Furthermore, the rate constant (Kh) at pH 5.4 (10.910 h⁻⁰^.^⁵) was higher than that detected at pH 7.4 (3.943 h⁻⁰^.^⁵). This observation reflected the rapid diffusion process at the acidic pH, consistent with the faster release of Cp observed at pH 5.4. These findings could further highlight the pH-sensitive nature of the BME NPs and the nanoparticles’ ability to release the Cp more efficiently in the acidic environment of tumors, where the pH is typically lower than in normal tissues.

Slower diffusion is desirable in normal tissues, as it suggests that the BME NPs are less likely to release the contained Cp under physiological conditions, thereby minimizing potential side effects and ensuring that more of the drug is retained within the nanoparticles until they reach the tumor site [46]. The Korsmeyer–Peppas model further elucidated the complexity of the release mechanism [47]. The release exponent (n) at pH 5.4 and pH 7.4 was 0.608 and 0.605, respectively, both indicating non-Fickian transport. This suggests that the release mechanism at both pH levels involved a combination of diffusion and matrix erosion or swelling. The slightly higher n value at pH 5.4 implies a greater contribution from erosion-related processes under acidic conditions, possibly due to the enhanced degradation or swelling of the nanoparticle matrix in the tumor-like environment [48]. The results indicated that the BME NPs could offer a pH-sensitive release mechanism, where the acidic conditions in the tumor microenvironment promote a more rapid and extensive release of Cp, potentially leading to improved therapeutic outcomes [42,43]. In contrast, the slower release at physiological pH suggests that the nanoparticles remain more stable in normal tissues, thereby minimizing systemic exposure and side effects. The combination of diffusion and matrix erosion as the release mechanisms could provide a controlled and sustained release profile that is desirable for targeted cancer therapy.

### 3.5. Cellular Uptake Assay

Cellular uptake of free Cp and Cp@CS/BME NPs was evaluated using HPLC to determine whether the incorporation of Cp into the BME NPs would enhance its accumulation in HT-29 and Caco-2 colon cancer cells. The cells were incubated with free Cp or Cp@CS/BME NPs for 4, 6, 12, and 24 h, and the cellular uptake was assessed at each timepoint.

**Figure 4:**
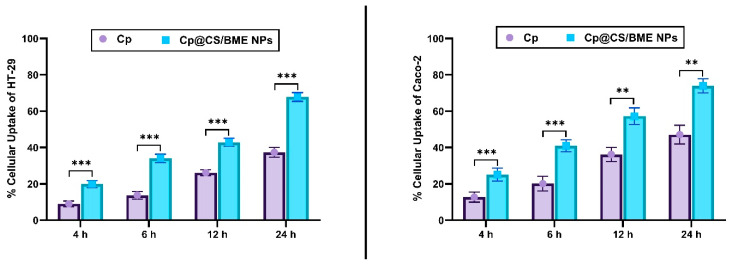
The uptake of Cp in its free or Cp@CS/BME NPs forms by HT-29 cells (left panel) and Caco-2 cells (right panel). Results represent the mean value ± SD (n = 3). The symbols *, **, *** indicate statistical significance from control (Cp) at *p*-values < 0.05, <0.01, and <0.001, respectively.

A significant increase in Cp accumulation was observed in both HT-29 and Caco-2 cells treated with Cp@CS/BME NPs compared to those treated with free Cp over the incubation periods (*p* < 0.01), as shown in Figure 4. Coating with CS, a positively charged molecule [49], could promote strong interactions with the negatively charged cancer cell surfaces [50]. Accordingly, the CS-coated nanoparticles (Cp@CS/BME NPs) were anticipated to show enhanced binding affinity or attraction towards cancer cells. Hence, this enhanced intracellular accumulation of Cp@CS/BME NPs could be attributed to the positive charges on the surface of the nanoparticles, which facilitate electrostatic interactions with the cancer cell surfaces, thereby improving cellular uptake [51].

The uptake percentages of Cp@CS/BME NPs by HT-29 cells at 4, 6, 12, and 24 h were 19.88 ± 1.9%, 34.01 ± 2.30%, 42.85 ± 2.20%, and 67.83 ± 2.41%, respectively. In comparison, the uptake percentages of Cp@CS/BME NPs by Caco-2 cells at the same timepoints were 25.16 ± 3.55%, 40.96 ± 3.29%, 57.26 ± 4.57%, and 74.01 ± 3.91%, respectively. These results indicate that the cellular uptake of Cp@CS/BME NPs was consistently higher in Caco-2 cells compared to HT-29 cells at all timepoints.

### 3.6. Cell Viability

**Figure 5:**
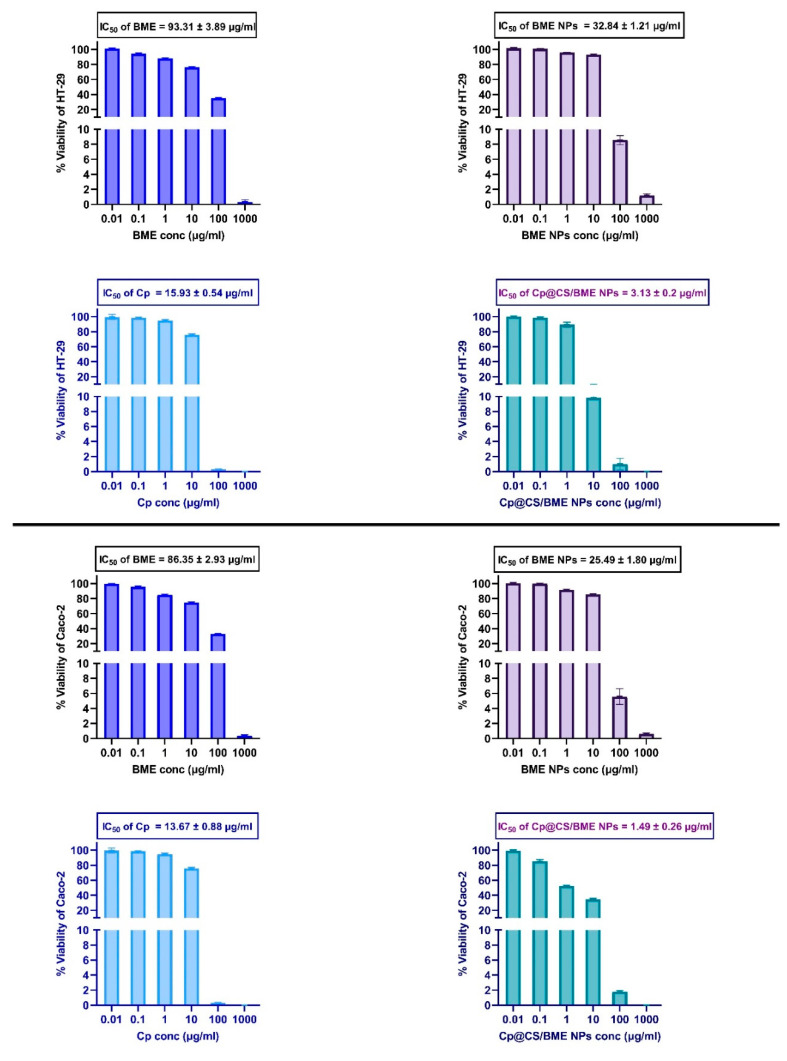
Dose–response curves showing the inhibitory effects of BME, BME NPs, Cp, and Cp@CS/BME NPs on the HT-29 cells (top panel) and caco-2 cells (bottom panel). Results represent the mean value ± SD (n = 3).

Cp is a broad-spectrum platinum-based drug employed in the treatment of colorectal carcinoma. However, its clinical effectiveness is often limited by challenges such as the development of drug resistance and systemic side effects [13]. This study focused on developing an innovative drug delivery system to minimize side effects and enhance the delivery to cancer cells. The proposed system comprised a Cp-loaded and CS-coated nanosized BME. This approach was based on previous studies highlighting the significance of each component in the system—specifically, the BME that had been previously reported to exhibit significant cytotoxic activity [52,53]. The use of natural CS macromolecules in nano drug delivery systems could offer several therapeutic benefits. For example, CS nanocomposites have been widely recognized for their safety, affordability, sustainability, biodegradability, biocompatibility, and high reactivity [54]. The cytotoxic activity of BME, BME NPs, Cp, and the novel Cp@CS/BME NPs against the colorectal cancer cell lines HT-29 and Caco-2 was evaluated using the SRB cellular viability assay. The half-maximal inhibitory concentration (IC50) values for each of the evaluated treatments are presented in Figure 5 and Table 5.

The cell viability studies carried out on HT-29 cells revealed interesting results. Treatment with BME alone exhibited the highest IC50 value of 93.31 ± 3.89 µg/mL. This value decreased to 32.84 ± 1.21 µg/mL upon treating the HT-29 cells with the nanosized BME, i.e., the BME NPs, demonstrating enhanced cytotoxicity compared to the free BME, likely due to improved cellular uptake of the BME in its nanosized forms. The IC50 of free Cp against HT-29 cells was 15.93 ± 0.54 µg/mL, which further decreased to 3.13 ± 0.2 µg/mL when incorporated into Cp@CS/BME NPs. Thus, the lowest IC50 value, indicating the highest potency, was observed for the Cp@CS/BME NPs treatment. This suggests a synergistic effect between Cp and BME when co-delivered via nanoparticles, enhancing the overall therapeutic efficacy.

The selectivity index (SI), which is defined as the ratio of IC50 values obtained from healthy cells to those obtained from cancer cells, reflects the safety of the tested anticancer drug towards healthy cells [55,56]. The SI values are detailed in Table 5. The SI values also support the observed findings; the SI for Cp alone was 2.02, whereas for Cp@CS/BME NPs it increased to 13, demonstrating a significantly improved safety profile for Cp@CS/BME NPs. Similarly, a previous study on MCF-7 breast cancer cells reported that loading BMR onto PLGA–PCL nanoparticles enhanced its cytotoxic activity compared to free *Boswellia sacra* oil [12].

A similar trend was observed in Caco-2 cells. The IC50 values for BME, BME NPs, Cp, and Cp@CS/BME NPs following incubation with Caco-2 cells were 86.35 ± 2.93 µg/mL, 25.49 ± 1.8 µg/mL, 13.67 ± 0.88 µg/mL, and 1.49 ± 0.26 µg/mL, respectively (Figure 5). The IC50 value for Cp@CS/BME NPs was approximately nine times lower than that of Cp, indicating a ninefold increase in potency and efficacy when Cp was loaded onto the Cp@CS/BME NPs.

These findings are consistent with the observations in HT-29 cells, where the nanoparticle formulations, particularly Cp@CS/BME NPs, demonstrated superior cytotoxicity compared to the free forms. The reduction in IC50 for Cp@CS/BME NPs in both cell lines highlights the potential of nanoparticle-mediated delivery systems in enhancing the efficacy of anticancer agents by facilitating improved cellular uptake and possibly overcoming resistance mechanisms.

Additionally, the SI of Cp was 2.36, which increased to 27.31 with Cp@CS/BME NPs (Table 5), demonstrating that Cp@CS/BME NPs could offer superior selectivity for Caco-2 cells and reduced toxicity to CCD 841 CoN normal cells.

The increased cytotoxicity observed with the Cp@CS/BME NPs treatment could be attributed to the nanoformulations’ ability to enhance the intracellular accumulation of Cp, as demonstrated in the cell uptake studies. One of the key factors contributing to the increased cellular uptake of Cp@CS/BME NPs was likely the presence of cationic CS in the nanoparticle formulation, which could enhance the electrostatic interaction with the anionic cell surface, leading to more efficient internalization of the nanoparticles by the cancer cells [33,57,58]. This enhanced cellular uptake, combined with the inherent cytotoxicity of both Cp and BME, likely underpins the significantly lower IC50 values observed for Cp@CS/BME NPs [11,12,53,59,60,61].

Interestingly, the IC50 of the Cp@CS/BME NPs treatment against Caco-2 cells was approximately half of that detected for HT-29 cells, reflecting higher cytotoxicity of Cp@CS/BME NPs towards Caco-2 cells compared to HT-29 cells. This finding aligned with those of cell uptake studies that showed higher uptake of Cp@CS/BME NPs by Caco-2 cells compared to HT-29 cells.

These findings highlight the synergistic potential of combining natural products such as BME with conventional chemotherapeutic agents in a nanoparticle-based delivery system. The lower IC50 values observed for Cp@CS/BME NPs suggest a promising therapeutic strategy for colorectal cancer, offering a potential avenue for reducing the Cp dosage while maintaining or even enhancing its anticancer efficacy, thereby minimizing the associated side effects. The role of cationic chitosan in enhancing cellular uptake further supports the use of such nanoparticle systems in targeted cancer therapy. Further in vivo studies are warranted to evaluate the therapeutic potential and safety profile of Cp@CS/BME NPs in colorectal cancer treatment.

### 3.7. Apoptosis Assay

The apoptotic effects of Cp were previously assessed against various cancer cell types, including colorectal cancer (HCT-116) [13], non-small-cell lung cancer (A549) [62], ovarian cancer (OVCA429) [63], and cervical cancer (SiHa) [64]. In the present study, an apoptosis assay using flow cytometric analysis was conducted to investigate the mechanisms of cell death in HT-29 and Caco-2 cells following 48 h of exposure to Cp@CS/BME NPs. Untreated cells were used as the control group for comparison.

**Figure 6:**
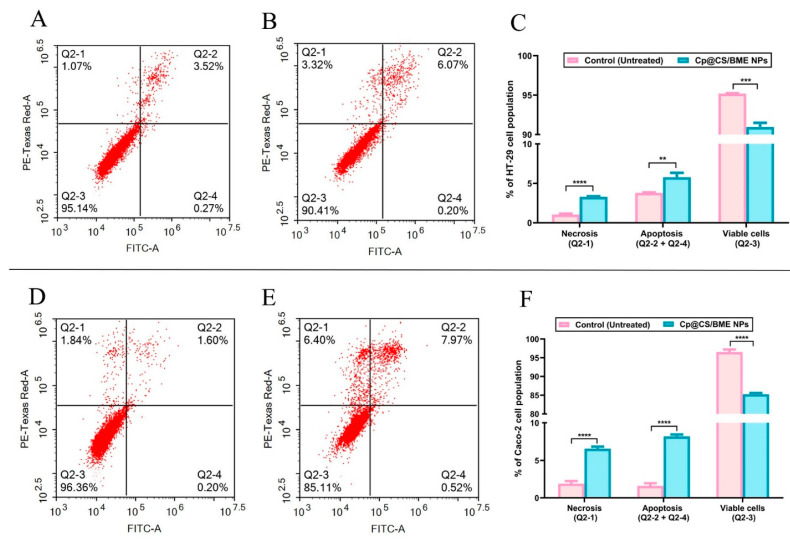
Apoptotic effects were observed in the HT-29 and Caco-2 cell lines following 48 h of exposure to Cp@CS/BME NPs. The top panel presents cytograms of Annexin V/propidium iodide-stained HT-29 cells, comparing untreated cells as the control group (**A**) with Cp@CS/BME NPs-treated cells (**B**). The corresponding bar graph (**C**) quantifies the percentages of necrotic, apoptotic, and viable HT-29 cells. The bottom panel depicts the cytograms for Annexin V/propidium iodide-stained Caco-2 cells, showing untreated control cells (**D**) and Cp@CS/BME NPs-treated cells (**E**). The bar graph (**F**) illustrates the proportions of necrotic, apoptotic, and viable Caco-2 cells. ** *p* < 0.01, *** *p* < 0.001, and **** *p* < 0.0001. Results represent the mean value ± SD (n = 3).

Incubation of HT-29 cells with Cp@CS/BME NPs at a dose of 3.13 µg/mL led to a significantly higher percentage of apoptotic cells (5.78 ± 0.56%) compared to the untreated control group (3.79 ± 0.08%) (*p* < 0.01) (Figure 6A–C). Additionally, treatment with Cp@CS/BME NPs resulted in an approximately threefold increase in the percentage of necrotic cells (3.28 ± 0.08%) relative to the untreated cells (1.03 ± 0.13%), with *p* < 0.0001. The percentage of viable HT-29 cells decreased significantly, from 95.18 ± 0.06% in the untreated group to 90.95 ± 0.55% in the Cp@CS/BME NPs-treated group (*p* < 0.001). Therefore, one can conclude that 48 h of incubation with the Cp@CS/BME NPs treatment at a concentration of 3.13 µg/mL increased the apoptosis and necrosis of HT-29 cells by 1.5- and 3-fold as compared to the untreated group, respectively. In comparison, previous work has shown similar results for the commercial drug oxaliplatin (oxp) upon application to HT-29 cells. The addition of oxp to HT-29 cells at a concentration of 20 µmol/L (or 7.9 µg/mL) resulted in about a 2.5-fold increase in the apoptotic quartiles as compared to the untreated group (42.28 % of cells in the apoptotic quartiles of the treated group vs. 16.5% of cells in the apoptotic quartiles of the untreated group) [65]. Moreover, oxp could increase the necrotic cell population from 3.26% to 5.75 %, which was an approximately 1.8-fold increase in necrosis [65]. Another FDA-approved antitumor drug, doxorubicin (dox), was reported to increase the cellular population in the late apoptosis quartile by 1.4-fold when applied at a dose of 100 μM (or 54.35 µg/mL). Thereupon, dox elevated the percentage of cells from 22.8% in the late apoptosis quartile of those treated with the free nanosized drug carrier DNA tetrahedron (control group) to 32.8% in the late apoptosis quartile of those treated with dox only [66]. A previous study showed that the application of 10 µmol/L (or 1.3 µg/mL) 5-fluorouracil (5-FU) resulted in a 9-fold increase in the apoptotic quartiles of HT-29 cells relative to untreated cells [67]. As such, the apoptotic effects of our Cp@CS/BME NPs on HT-29 cells are superior to those of the commercial drugs oxp and dox but less than that of 5-FU.

For Caco-2 cells, treatment with Cp@CS/BME NPs at a dose of 1.49 µg/mL induced a markedly higher percentage of necrotic cells (6.57 ± 0.29%) compared to the control group (1.89 ± 0.36%), with *p* < 0.0001 (Figure 6D–F). The percentage of apoptotic Caco-2 cells increased by approximately fivefold (8.2 ± 0.27%) relative to the untreated cells (1.58 ± 0.37%), with *p* < 0.0001. Moreover, the percentage of viable Caco-2 cells significantly declined (*p* < 0.0001), from 96.53 ± 0.69% in the untreated group to 85.23 ± 0.36% in the Cp@CS/BME NPs-treated group. Set side by side with other commercial drugs, previous work has shown that the percentage of Caco-2 cells in early apoptosis was 12% in untreated cells (control) and significantly increased to 21% with 5 μM (or 1.98 µg/mL) of sole oxp treatment [68]. This means that oxp increased the apoptosis of Caco-2 cells by approximately 1.8-fold relative to their untreated counterparts [68]. Surprisingly, the application of dox at a dose of 1 µM (or 0.54 µg/mL) to Caco-2 cells elevated the percentage of cells in the late apoptosis quartile to 34%, as opposed to 1.1% in the control group (untreated cells) [69]. This, in turn, indicates that dox induced a 31-fold increase in apoptosis progression [69]. Meanwhile, the incubation of Caco-2 cells with 47 µM (or 6.1 µg/mL) 5-FU increased the percentage of apoptotic cells by approximately 3.6-fold relative to the untreated counterparts [70]. The percentage displayed by the apoptotic cells was estimated as 14% in the untreated group vs. 50% in the 5-FU-treated group [70]. All these findings emphasize the pronounced apoptotic effects of our novel formulation (Cp@CS/BME NPs) on Caco-2 cells, which outrivaled those of oxp and 5-FU.

Collectively, these observations demonstrate the ability of the Cp@CS/BME NPs treatment to induce marked apoptotic effects in the HT-29 and Caco-2 cancer cell lines, surpassing the effects of commercial drugs of the same class, such as oxp.

### 3.8. Cell-Cycle Analysis

**Figure 7:**
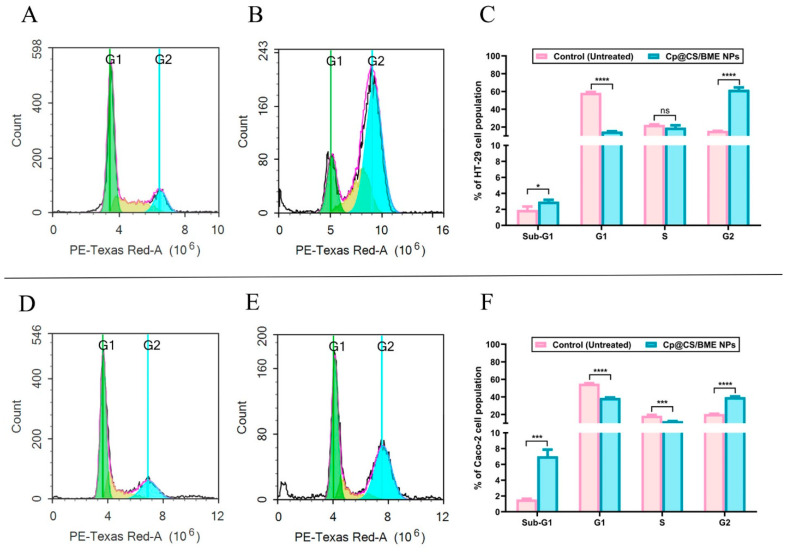
Cell-cycle analysis of HT-29 and Caco-2 cells treated with Cp@CS/BME NPs. The top panel presents cytograms of untreated (control) HT-29 cells (**A**) and HT-29 cells treated with Cp@CS/BME NPs (**B**), accompanied by a bar graph illustrating the cell-cycle distribution of HT-29 cells (**C**). The bottom panel shows the cytograms of untreated (control) Caco-2 cells (**D**) and Caco-2 cells treated with Cp@CS/BME NPs (**E**), along with a bar graph showing the cell-cycle distribution of Caco-2 cells (**F**). * *p* < 0.05, ** *p* < 0.01, *** *p* < 0.001, and **** *p* < 0.0001. Results represent the mean value ± SD (n = 3).

The cell-cycle kinetics was assessed for both HT-29 and Caco-2 cancer cells after 48 h of exposure to Cp@CS/BME NPs at their respective IC50 concentrations (Figure 7). The fractional DNA content of apoptotic cells can typically be observed due to the staining procedure, which involves the extraction of fragmented DNA of low molecular weight. Apoptotic cells often shed DNA by releasing apoptotic bodies, resulting in a partial retention of DNA within these cells. This phenomenon is represented in DNA content frequency cytograms as a peak, referred to as the ‘sub-G1’ peak [71]. In HT-29 cells, treatment with Cp@CS/BME NPs led to a significant increase in the sub-G1 population percentage (2.97 ± 0.23%) compared to untreated cells (1.93 ± 0.43%), with a *p*-value < 0.05, as shown in Figure 7A–C. A similar trend, with a more pronounced effect, was observed in Caco-2 cells, where the sub-G1 population percentage increased from 1.56 ± 0.10% in untreated cells to 7.03 ± 0.84% in Cp@CS/BME NPs-treated cells, with a *p*-value < 0.001, as depicted in Figure 7D–F.

During the synthesis phase, referred to as the ’S phase’, cells duplicate their DNA content, producing identical copies of their genetic material [72]. Our findings revealed a significant reduction in the percentage of Caco-2 cells in the S phase, decreasing from 18.56 ± 1.04% in untreated cells to 12.3 ± 0.13% in cells treated with Cp@CS/BME NPs, with a *p*-value < 0.001 (Figure 7D–F). In contrast, treatment with Cp@CS/BME NPs did not produce a notable effect on the S phase of HT-29 cells (Figure 7A–C).

Following the S phase, cells briefly enter the G2 phase, during which they prepare for mitosis (M) [73]. The transition from G2 to M is typically regulated by CDK1. However, when these regulatory mechanisms are disrupted, it can lead to uncontrolled cell division and cancer development. Our results demonstrated that Cp@CS/BME NPs effectively induced cell accumulation in the G2 phase and inhibited the G2/M transition in both HT-29 and Caco-2 cells. The untreated HT-29 cells exhibited 15.66 ± 0.12% of the cellular population in the G2 phase, whereas Cp@CS/BME NPs-treated HT-29 cells showed a substantial increase to 61.91 ± 2.64% in the G2 phase. This highly significant difference (*p* < 0.0001) highlights the ability of Cp@CS/BME NPs to sequester cells in the G2 phase (Figure 7A–C). A similar trend was observed in Caco-2 cells, with 20.56 ± 0.29% of the cellular population in the G2 phase in the untreated control group, compared to 39.70 ± 0.87% in Cp@CS/BME NPs-treated cells, showing a significant difference (*p* < 0.0001) (Figure 7D–F).

The results demonstrated the effects of treatment with Cp@CS/BME NPs on the cell cycle of both colorectal cancer cell types, revealing a significant sequestration of cells in the sub-G1 and G2 phases, along with a notable ability to limit the G2/M transition. These findings are consistent with those of a previous study that demonstrated the ability of free Cp treatment to accumulate the colon adenocarcinoma (CT-26) cells in the G2 phase and reduce the G2/M transition [74]. The cell-cycle analysis results could be linked to Cp@CS/BME NPs’ capability to boost the cytotoxic effects. This outcome might stem from the increased cellular uptake of Cp enabled by the CS-coated nanoformulation, along with the combined cytotoxic effects provided by the BME component [33,59].

### 3.9. Gene Expression

The Bcl-2 protein family plays a crucial role in regulating programmed cell death. This family comprises several pro-apoptotic proteins, including BID (BH3-interacting domain death agonist), BIK (BCL2-interacting killer), and PMAIP-1 (phorbol-12-myristate-13-acetate-induced protein 1), as well as anti-apoptotic proteins such as Bcl-2 (B-cell lymphoma 2) [75]. These genetic markers have been extensively investigated across various cancer types. Alterations in their expression levels or genetic mutations can significantly influence cancer progression and treatment efficacy. For example, elevated expression of Bcl-2 or reduced expression of BID, BIK, or PMAIP-1 has been associated with increased chemotherapy resistance and poorer prognosis in numerous tumors [76,77,78].

**Figure 8:**
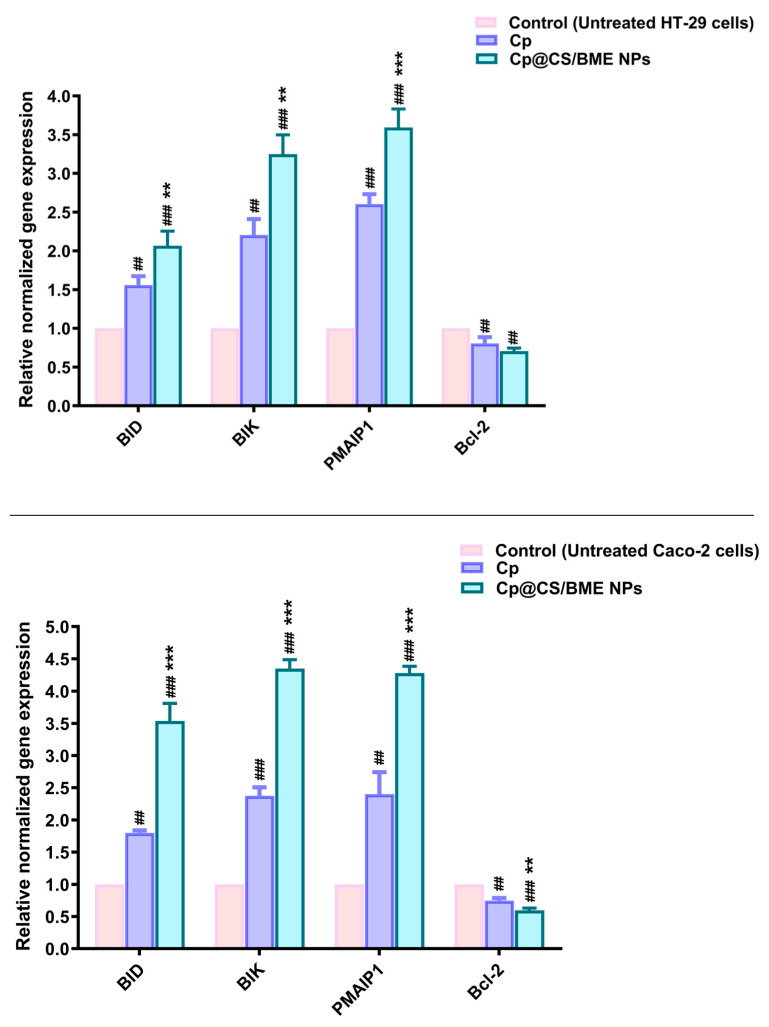
RT-qPCR analysis of target genes in HT-29 (top panel) and Caco-2 cells (bottom panel) following 48 h incubation with Cp or Cp@CS/BME NPs. Gene expression levels are normalized to β-actin and presented as the mean ± SD of three independent experiments; (#) and (*) indicate statistical significance compared to the control and Cp-treated groups, respectively. ** *p* < 0.01, ^##^ *p* < 0.01, *** *p* < 0.001 and ^###^ *p* < 0.001.

BID, a pro-apoptotic protein, facilitates the release of cytochrome c from the mitochondria, thereby promoting cell death. It acts as a critical link between death receptor signaling and the mitochondrial apoptotic pathway [79,80]. Our results indicated that, in HT-29 cells, treatment with Cp and Cp@CS/BME NPs led to approximately 1.6- and 2.1-fold increases, respectively, in the relative normalized expression of the BID gene (Figure 8). In Caco-2 cells, Cp alone resulted in about a 1.8-fold increase in BID gene expression, whereas Cp@CS/BME NPs treatment induced a more substantial increase (approximately 3.5-fold) in BID expression (Figure 8).

BIK is a pro-apoptotic protein that counteracts the anti-apoptotic effects of Bcl-2, thereby promoting cell death. Additionally, BIK can initiate mitochondrial apoptosis by transporting calcium from the endoplasmic reticulum to the mitochondria and reorganizing the mitochondrial cristae [81]. In the present study, treatment with Cp and Cp@CS/BME NPs in HT-29 cells led to significant increases in BIK expression, with approximately 2.2- and 3.2-fold elevations, respectively (Figure 8). Similarly, in Caco-2 cells, treatments with Cp and Cp@CS/BME NPs resulted in about 2.4- and 4.3-fold increases in BIK expression, respectively (Figure 8).

PMAIP-1, also referred to as NOXA, is a protein that binds to and inhibits pro-survival members of the Bcl-2 family, thereby triggering the intrinsic pathway of apoptosis [82,83]. In the current study, the Cp and Cp@CS/BME NPs treatments led to approximately 2.6- and 3.6-fold increases in the relative normalized expression of the PMAIP-1 gene in HT-29 cells, respectively (Figure 8). Similarly, in Caco-2 cells, the Cp and Cp@CS/BME NPs treatments resulted in approximately 2.4- and 4.3-fold increases in PMAIP-1 gene expression, respectively.

The significantly increased expression of pro-apoptotic genes observed with the Cp@CS/BME NPs treatment, compared to free Cp, could be due to the nanoformulation’s capacity to enhance Cp uptake due to its CS coating, as demonstrated by cell uptake studies, as well as the synergistic cytotoxic effects induced by the BME [52,53,59,84].

Conversely, Bcl-2 is an anti-apoptotic protein that contributes to cell survival and prevents cell death. It inhibits the release of cytochrome c from the mitochondria and prevents the subsequent activation of apoptotic proteins [8]. Our results demonstrated that treatment with Cp and Cp@CS/BME NPs reduced Bcl-2 gene expression in HT-29 cells by approximately 20% and 30%, respectively (Figure 8). A more pronounced effect was observed in Caco-2 cells, where the Cp and Cp@CS/BME NPs treatments led to reductions of 25% and 40% in Bcl-2 expression, respectively.

This study highlights the significant ability of Cp@CS/BME NPs to upregulate apoptotic genes (BID, BIK, PMAIP-1) and downregulate the anti-apoptotic gene Bcl-2, surpassing the effects observed in both untreated and free-Cp-treated groups. These findings highlight the enhanced cytotoxic and apoptotic efficacy of Cp@CS/BME NPs, rendering them a promising therapeutic approach for colorectal carcinoma.

## 4. Conclusions

This study successfully demonstrates the synergistic potential of using nanoparticles formulated with a naturally derived BME to enhance the therapeutic efficacy of Cp for colorectal cancer treatment. The pH-sensitive release profile of Cp@CS/BME nanoparticles, with increased drug release in acidic environments, suggests a targeted approach that may improve treatment outcomes in tumor tissues. The in vitro studies revealed that these nanoparticles significantly enhance the cellular uptake and cytotoxic activity of Cp in the HT-29 and Caco-2 colorectal cancer cell lines. Moreover, the enhanced induction of apoptosis and cell-cycle alterations observed with Cp@CS/BME NPs highlight their effectiveness in promoting cancer cell death. The observed downregulation of the anti-apoptotic gene Bcl-2 and upregulation of the pro-apoptotic genes Bax, PUMA, and BID further support the therapeutic potential of the nanoformulation. Future studies would benefit from the assessment of Cp@CS/BME NPs’ efficacy in vivo to further evaluate their therapeutic potential and safety profile. Overall, this study demonstrates the potential of naturally derived ingredient-based nanoparticles to optimize chemotherapeutic efficacy and reduce side effects, offering a promising strategy for advanced cancer nanomedicine in colorectal cancer therapy.

## Figures and Tables

**Table 1:** Primers used in RT-qPCR.

Primer ID	Primer Sequence (5′-3′)
β-actin F	CACCATTGGCAATGAGCGGTTC
β-actin R	AGGTCTTTGCGGATGTCCACGT
BID F	TGGTGTTTGGCTTCCTCCAA
BID R	GAATCTGCCTCTATTCTTCCC
BIK F	GAGACATCTTGATGGAGACC
BIK R	TCTAAGAACATCCCTGATGT
Noxa (PMAIP1) F	AGCAGAGCTGGAAGTCGAGTGTG
Noxa (PMAIP1) R	TGATGCAGTCAGGTTCCTGAGC
Bcl-2 F	ATCGCCCTGTGGATGACTGAGT
Bcl-2 R	GCCAGGAGAAATCAAACAGAGGC

**Table 2:** Identified peaks in the LC-MS spectrum of the BME.

Peak	Rt	Assignment	Precursor Ion (*m*/*z*)	Molecular Formula	Productions MS/MS	Class
1.	2.65	Quinic acid	191.0455	C_7_H_12_O_6_	191, 173, 147, 127, 109, 93, 87, 85, 67	Organic acid
2.	2.77	D-(+)-Trehalose	341.1519	C_12_H_22_O_11_	341, 211, 179, 89	Disaccharide
3.	2.84	Quinic acid	191.0819	C_7_H_12_O_6_	191, 173, 147, 127, 109, 93, 87, 85, 67	Organic acid
4.	3.11	Ureidosuccinic acid	174.9804	C_5_H_8_N_2_O_5_	175, 132	Organic acid
5.	3.18	Quinic acid	191.0457	C_7_H_12_O_6_	191, 173, 147, 127, 109, 93, 87, 85, 67	Organic acid
6.	5.64	Methylmalonic acid	117.0362	C_4_H_6_O_4_	117, 116, 115	Organic acid
7.	10.56	Oxalacetic acid	131.0534	C_4_H_4_O_5_	131, 130, 129, 128	Organic acid
8.	10.64	Neochlorogenic acid	353.1439	C_16_H_18_O_9_	353, 259, 191, 180 135, 134	Phenolic acid
9.	10.79	Tridecanoyl-sn-glycero-3-phosphate	367.2089	C_21_H_44_NO_7_P	367, 311, 215, 153, 123	Phospholipid
10.	10.83	Neochlorogenic acid	353.0947	C_16_H_18_O_9_	353, 259, 191, 180 135, 134	Phenolic acid
11.	11.05	Neochlorogenic acid	353.1336	C_16_H_18_O_9_	353, 259, 191, 180 135, 134	Phenolic acid
12.	11.24	2-(2-Hydroxyethoxy)phenol	153.0406	C_8_H_10_O_3_	153, 150, 109, 91	Phenol
13.	12.80	Gabapentin-related compound E	185.1069	C_9_H_14_O_4_	185, 142, 141, 123, 99, 81, 71, 57	Organic acid
14.	14.54	6-Fluoro-4-hydroxycoumarin	381.1810	C_9_H_5_FO_3_	381, 309, 180, 179, 136, 135, 94	Hydroxycoumarin
15.	15.07	2′-Hydroxy-4′-methoxyacetophenone	165.0776	C_9_H_10_O_3_	165, 150, 122, 108, 71	Phenol
16.	15.22	Madecassic acid	503.4005	C_30_H_48_O_6_	503, 499, 443, 371, 248	Triterpene
17.	15.71	2′-Hydroxy-4′-methoxyacetophenone	165.0785	C_9_H_10_O_3_	165, 150, 122, 108, 71	Phenol
18.	16.01	Madecassic acid	503.3997	C_30_H_48_O_6_	503, 443, 399	Triterpene
19.	16.35	Maslinic acid	471.3008	C_30_H_48_O_4_	471, 427, 397, 353, 314, 263, 217, 189, 145, 113	Triterpene
20.	16.47	4-Androsten-17.beta-ol-3-one sulfate	367.2594	C_19_H_27_O_5_S	367, 287, 243, 85	Steroid
21.	17.15	7,7-Dimethyl-(5Z,8Z)-eicosadienoic acid	335.2658	C_22_H_40_O_2_	335, 291	Fatty acid
22.	17.26	N-2-Hydroxyethylpiperazine	251.1983	C_6_H_14_N_2_O	251	Alkaloid
23.	17.34	3-Phenylbutyric acid	163.0985	C_10_H_12_O_2_	163, 148, 134	Organic acid
24.	17.34	Thomboxane B3	367.2588	C_20_H_32_O_6_	367, 352, 331, 251, 230, 170, 169, 122, 97	Eicosanoid
25.	17.60	(+)-trans-Chrysanthemic acid	167.1306	C_10_H_16_O_2_	167, 133, 109	Monoterpene
26.	17.87	cis-4,10,13,16-Docosatetraenoic acid	331.2335	C_22_H_36_O_2_	331, 288, 287, 236, 83	Fatty acid
27.	17.98	Genkwanin	283.0990	C_16_H_12_O_5_	283, 268, 251, 179, 135, 79	Flavonoid
28.	22.33	4-Chloro-alpha-(4-chlorophenyl)-benzeneacetic acid	279.2334	C_14_H_9_Cl_2_O_2_^-^	279, 236, 235, 199, 183, 153, 134, 97, 71	Organic acid
29.	22.90	11-Keto-beta-boswellic acid	469.3891	C_30_H_46_O_4_	469, 452, 407, 391, 376, 271, 61	Triterpene
30.	23.58	Trihydroxycholestanoic acid	449.3703	C_27_H_46_O	449, 327	Bile acid
31.	23.70	2,2′-Methylene-bis(6-tert-butyl-4-methylphenol)	339.2758	C_23_H_32_O_2_	339, 327, 165, 164, 163, 147	Phenol
32.	24.38	3beta,7alpha-Dihydroxy-5-cholestenoic acid	431.3579	C_27_H_44_O_4_	431	Bile acid
33.	24.53	Maslinic acid	471.4052	C_30_H_48_O_4_	471	Triterpene
34.	26.57	3-Acetyl-11-keto-beta-boswellic acid	511.4039	C_32_H_48_O_5_	511, 60, 59	Triterpene
35.	27.37	3-Acetyl-11-keto-beta-boswellic acid	511.4054	C_32_H_48_O_5_	511, 60, 59	Triterpene

**Table 3:** The average particle size, PDI, zeta potential, and EE% of the engineered BME NPs and Cp@CS/BME NPs. Data are presented as the mean ± SD; n = 3.

Samples	Average Particle Size (nm)	PDI	EE (%)
BME NPs	120.10 ± 5.10	0.11 ± 0.33	-
Cp@CS/BME NPs	160.20 ± 4.60	0.14 ± 0.04	86.50 ± 2.80

**Table 4:** The rate constants and correlation coefficients (R^2^) for Cp release at pH 5.4 or 7.4 from BME NPs were determined by fitting them to different release models.

		pH 5.4	pH 7.4
**Zero-order**	*k*_0_	1.412	0.497
	R^2^	0.797	0.731
**First-order**	*k*_1_	0.041	0.040
	R^2^	0.539	0.385
**Higuchi matrix**	*Kh*	10.910	3.943
	R^2^	0.970	0.937
**Korsmeyer–Peppas**	n	0.608	0.605
	R^2^	0.640	0.757
**Hixson–Crowell**	κ t	0.007	0.002
	R^2^	0.883	0.756

**Table 5:** The cytotoxic activity of BME, BME NPs, Cp, and Cp@CS/BME NPs against the CCD 841 CoN normal colon epithelial cell line and the SI for HT-29 and Caco-2 cancer cell lines.

Sample	CC_50_^a^	SI for HT-29 Cancer Cells ^b^	SI for Caco-2 Cancer Cells ^c^
BME	296.03 ± 3.99	3.17	3.42
BME NPs	190.00 ± 6.98	5.79	7.45
Cp	32.21 ± 1.97	2.02	2.36
Cp@CS/BME NPs	40.69 ± 2.29	13.00	27.31

^a^ Cytotoxic activity against CCD 841 CoN normal cell line (μg/mL); presented as average ± SD (n = 3). ^b^ Selectivity index calculated as CC_50_/IC_50_ against HT-29 cells. ^c^ Selectivity index calculated as CC_50_/IC_50_ against Caco-2 cells.

## Data Availability

The original contributions presented in this study are included in the article/Supplementary Materials; further inquiries can be directed to the corresponding authors.

## Supplementary Materials

The following supporting information can be downloaded at: https://www.mdpi.com/article/10.3390/pharmaceutics16101282/s1, Figure S1: Size distribution curves of (A) BME NPs and (B) Cp@CS/BME NPs.
