# Supplementary material for: Synergistic Enhancement of Carboplatin Efficacy through pH-Sensitive Nanoparticles Formulated Using Naturally Derived Boswellia Extract for Colorectal Cancer Therapy
Source: Pharmaceutics. 2024 Sep 30;16(10):1282. doi: 10.3390/pharmaceutics16101282 (PMC11510476; doi:10.3390/pharmaceutics16101282)
Supplement: Supplementary file 1 [file pharmaceutics-16-01282-s001.zip › pharmaceutics-3208922-supplementary.pdf]

Synergistic Enhancement of Carboplatin Efficacy through pH-Sensitive Nanoparticles Formulated Using Naturally Derived Boswellia Extract for Colorectal Cancer Therapy

Sherif Ashraf Fahmy [1,2,*,†], Nada K. Sedky [3,†], Hatem A. F. M. Hassan [4,5], Nour M. Abdel-Kader [3,6], Noha Khalil Mahdy [5], Muhammad Umair Amin [2], Eduard Preis [2] and Udo Bakowsky [2,*]

[1] Department of Chemistry, School of Life and Medical Sciences, University of Hertfordshire Hosted by Global Academic Foundation, R5 New Garden City, New Administrative Capital, Cairo 11835, Egypt

[2] Department of Pharmaceutics and Biopharmaceutics, University of Marburg, Robert-Koch-Str. 4, 35037 Marburg, Germany; muhammad.umairamin@pharmazie.uni-marburg.de (M.U.A.); eduard.preis@pharmazie.uni-marburg.de (E.P.)

[3] Department of Biochemistry, School of Life and Medical Sciences, University of Hertfordshire Hosted by Global Academic Foundation, R5 New Garden City, New Administrative Capital, Cairo 11835, Egypt; nadasedky22@gmail.com (N.K.S.); nmohamed@gaf.edu.eg (N.M.A.-K.)

[4] Medway School of Pharmacy, Universities of Kent and Greenwich, Chatham Maritime, Kent ME4 4TB, UK; hatem.hassan@pharma.cu.edu.eg

[5] Department of Pharmaceutics and Industrial Pharmacy, Faculty of Pharmacy, Cairo University, Cairo 11562, Egypt; nkmahdy@gmail.com

[6] Department of Biochemistry, Faculty of Science, Ain Shams University, Cairo 11566, Egypt

[*] Correspondence: sheriffahmy@aucegypt.edu (S.A.F.); ubakowsky@aol.com (U.B.)

[†] These authors contributed equally to this work.

Supplementary Material

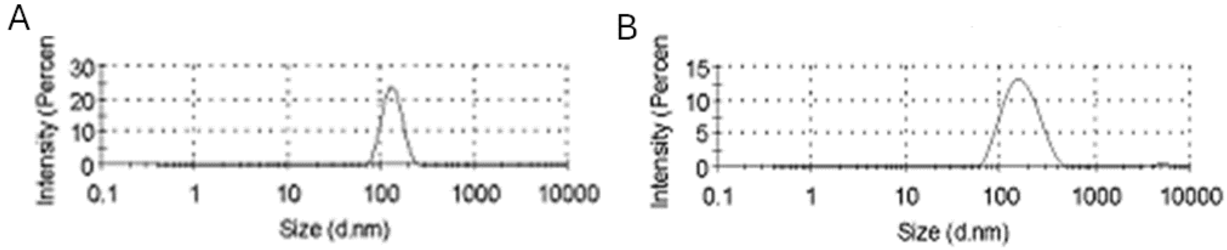

Figure S1. Size distribution curves of (**A**) BME NPs and (**B**) Cp@CS/BME NPs.
